# Treatment of acute diverticulitis laparoscopic lavage vs. resection (DILALA): study protocol for a randomised controlled trial

**DOI:** 10.1186/1745-6215-12-186

**Published:** 2011-08-01

**Authors:** Anders Thornell, Eva Angenete, Elisabeth Gonzales, Jane Heath, Per Jess, Zoltan Läckberg, Henrik Ovesen, Jacob Rosenberg, Stefan Skullman, Eva Haglind

**Affiliations:** 1 Department of Surgery, Alingsås Hospital, Södra Ringatan, Alingsås, 441 83, Sweden; 2 Department of Surgery, Sahlgrenska University Hospital/Östra, Göteborg, 413 45, Sweden; 3 Department of Surgery, Roskilde University Hospital, Kögevej 7-13, Roskilde, 4000, Denmark; 4 Department of Surgery, NU Hospital Organisation, Trollhättan, 461 85, Sweden; 5 Department of Surgery, Herlev University Hospital, Herlev Ringvej 75, Herlev, 2730, Denmark; 6 Department of Surgery, Skövde Hospital/KSS, Skövde, 541 85, Sweden

## Abstract

**Background:**

Perforated diverticulitis is a condition associated with substantial morbidity. Recently published reports suggest that laparoscopic lavage has fewer complications and shorter hospital stay. So far no randomised study has published any results.

**Methods:**

DILALA is a Scandinavian, randomised trial, comparing laparoscopic lavage (LL) to the traditional Hartmann's Procedure (HP). Primary endpoint is the number of re-operations within 12 months. Secondary endpoints consist of mortality, quality of life (QoL), re-admission, health economy assessment and permanent stoma. Patients are included when surgery is required. A laparoscopy is performed and if Hinchey grade III is diagnosed the patient is included and randomised 1:1, to either LL or HP. Patients undergoing LL receive > 3L of saline intraperitoneally, placement of pelvic drain and continued antibiotics. Follow-up is scheduled 6-12 weeks, 6 months and 12 months. A QoL-form is filled out on discharge, 6- and 12 months. Inclusion is set to 80 patients (40+40).

**Discussion:**

HP is associated with a high rate of complication. Not only does the primary operation entail complications, but also subsequent surgery is associated with a high morbidity. Thus the combined risk of treatment for the patient is high. The aim of the DILALA trial is to evaluate if laparoscopic lavage is a safe, minimally invasive method for patients with perforated diverticulitis Hinchey grade III, resulting in fewer re-operations, decreased morbidity, mortality, costs and increased quality of life.

**Trial registration:**

British registry (ISRCTN) for clinical trials ISRCTN82208287http://www.controlled-trials.com/ISRCTN82208287

## Background

Complicated diverticulitis sometimes requires emergency surgery with considerable morbidity [1]. It is classified by severity according to the Hinchey grading scale, where I and II represent contained abscesses, and III and IV are cases with perforated colon and purulent (III) or faecal (IV) leakage [2]. The traditional treatment for the Hinchey III and IV has been open surgery with resection of the affected segment, blind closure of the distal resection line and a diverting colostomy i.e. Hartmann's procedure [3-6]. Another option is resection with primary anastomosis of the colon [3]. A retrospective study at the Sahlgrenska University Hospital, Gothenburg studied all patients (n = 106) admitted and operated for complicated diverticulitis between 2003 and 2008 [7]. Eighteen percent underwent at least one re-operation during their first admission, and the mean length of hospital stay was 17 (1-111) days. Mortality was 6%, not different from similar studies [2,6]. The number of complications indicated considerable suffering, morbidity and resource consumption [7]. Only 56% of patients operated with Hartmann's procedure later underwent surgery for stoma reversal [7]. Other studies have shown that the reversal of Hartmann's Procedure alone has a morbidity rate of 20% (3-39) and mortality of 1-6%[8,9].

Several recently published reports suggest a new principle for the treatment of perforated diverticulitis, consisting of laparoscopy, lavage and drainage without colon resection [10-16]. One prospective study including 92 patients with laparoscopic lavage showed morbidity and mortality rates of 4% and 3%, respectively. No re-intervention was needed. Eighty-nine patients recovered without morbidity and resumed oral intake after 2 days (1-9) and were discharged after 8 days (7-19)[17].

Janson et al [17] have shown that laparoscopic resection for colon cancer results in a higher quality of life compared to open surgery, and although there is little evidence indicating laparoscopy being more cost-effective than open surgery [18], a recent Health Technology Assessment suggests that there are economical benefits to lavage of perforated purulent diverticulitis, due to decreased surgical measures and shorter length of hospital stay [19]. With this in consideration there are several factors in favour of the new treatment, laparoscopy and lavage.

Randomised studies are required before laparoscopic lavage can be proven to be the therapy of choice in perforated diverticulitis Hinchey grade III [19]. This is a description of a randomized trial for complicated diverticulitis initiated in Scandinavia.

## Methods

### Study Objective

The DILALA-trial is a randomised trial comparing laparoscopic lavage to Hartmann's Procedure, 1:1, as treatment for acute perforated diverticulitis.

### Endpoints

Primary endpoint is number of re-operations within 12 months from the initial emergency operation.

Secondary endpoints include re-admissions, postoperative wound or deep infections, postoperative thrombosis, hernia, bowel obstruction requiring hospitalisation or operation, other complications, total length of hospital stay (for diverticulitis and complications) during 12 months, quality of life, health economy analysis, mortality within 30 days of the primary operation, mortality within 12 months, permanent stoma, re-admissions and re-operations registered in the hospital database at 24 months.

### Inclusion Criteria

Inclusion criteria to diagnostic laparoscopy

• Clinical symptoms (left lower quadrant pain, peritonitis)

• Elevated body temperature

• Elevated C-reactive protein and leukocyte count.

• Radiology showing signs of free gas and/or intraabdominal fluid

• Emergency surgery decided by the attending surgeon

• Possible to operate in regard to concomitant disease

• Informed consent

### Randomisation Criteria

• Hinchey grade III at diagnostic laparoscopy, i.e. free fluid

### Excluded From Randomization

• Hinchey grade I - II at laparoscopy i.e. no free fluid

• Hinchey grade IV at laparoscopy, i.e. gross faecal contamination

• Other pathology than diverticulitis diagnosed as explanation of peritonitis

The Hinchey grading [2]

**Stage Ia: **Phlegmonae

**Stage Ib: **Diverticulitis with peri-colic or mesenteric abscess

**Stage II: **Diverticulitis with walled-off pelvic abscess

**Stage III: **Diverticulitis with generalised purulent peritonitis

**Stage IV: **Diverticulitis with generalised faecal peritonitis

### External Validity

All patients with acute diverticulitis considered for emergency surgery are registered in the "screening log" at each participating centre. Patients who do not meet the inclusion criteria, as well as included but not randomised patients or patients excluded after randomisation are registered.

### Randomisation and Surgical Procedure

Patients are considered for inclusion when surgery is required. The operation starts with a diagnostic laparoscopy. All four quadrants of the abdomen must be visualised to ascertain no other or concomitant pathology. Patients are randomised when the diagnosis of diverticulitis Hinchey grade III has been confirmed. Randomisation is 1:1 to laparoscopic lavage and Hartmann's Procedure. Hinchey grade I, II and IV are excluded from randomisation and treated according to local guidelines.

Patients randomised to lavage receive at least 3L of body-temperature, until return of clear fluid. A passive drainage is placed in the pelvis for at least 24 h, and antibiotics are continued postoperatively according to local guidelines.

**Figure 1:**
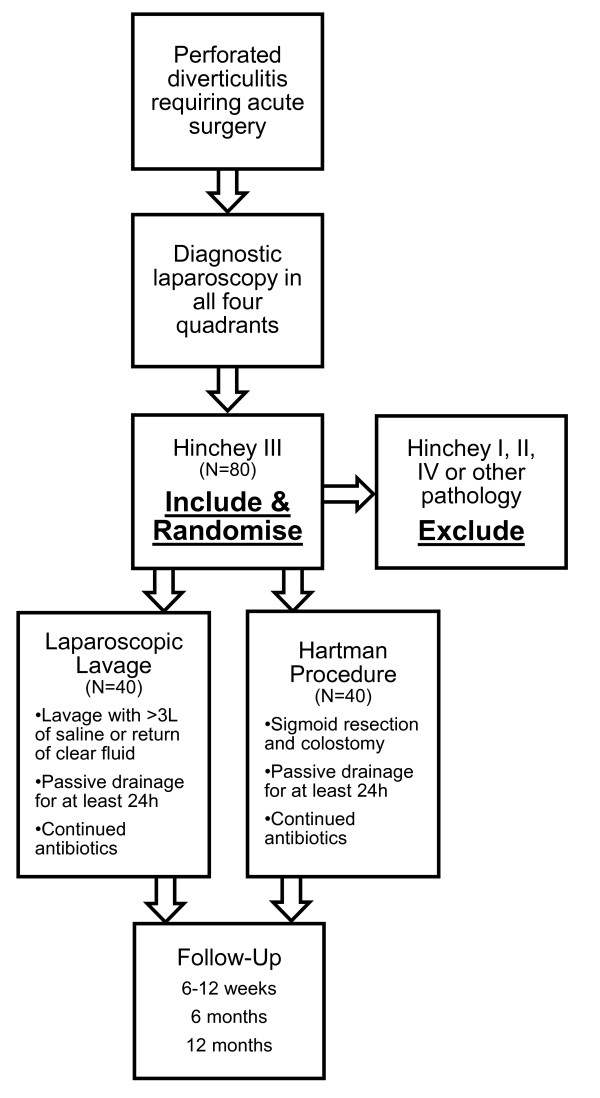
**Inclusion: Flow-chart**.

Patients randomised to Hartmann's Procedure are converted to open surgery followed by resection of the inflamed part of the colon and a diverting colostomy. A passive drainage is placed in the pelvis for at least 24 h, and antibiotics are continued postoperatively according to local guidelines (Figure 1).

All surgical specimens undergo histological examination. If the histological findings verify a pathogenesis other than diverticulitis, the patient will be excluded.

### Follow-Up

All follow-ups include haemoglobin, C-reactive protein, leukocyte count, presence of a stoma, re-admittance/s, re-operation/s. Clinical follow-up after discharge is scheduled at a minimum of 6-12 weeks, 6 months and 12 months. Patients randomised to the HP with planned stoma reversal are scheduled to have a follow-up at least 6-12 weeks following this procedure, regardless of the earlier follow-up plan from the primary operation. All patients undergo colonoscopy, computer tomography colonography or double contrast barium enema, within 12 months.

### Health Related Quality of Life

All patients are asked to fill out a quality of life form on the day of discharge, 6 months and 12 months. This form includes parts of EQ5D, SF 36 [20] EORTC-C30 [17] and -CR38 [21]. The questions are focusing on bowel symptoms, stoma care, activities of daily living, health economic status and bowel related episodes requiring re-admittance or re-operation.

### Health Economics Assessment

A health economic analysis will be performed based on the information collected in the clinical record forms (CRF), based on the model presented by Björholt et al [22]. The model will be used in combination with sensitivity calculations to ascertain robust results.

### Data Collecting and Monitoring

The surgeon will fill out the CRF for the operation, and at each follow-up. The hospitalisation CRF is filled out by a nurse. Quality of life forms are filled out by the patient at discharge and at each follow-up. All CRFs and quality of life forms are returned to the trial coordinating centre at Sahlgrenska University Hospital, SSORG. All data from CRFs and quality of life forms are stored on a server, and kept within the Sahlgrenska University Hospital database. Only the principal investigator and the deputy principal investigator can by mutual consent extract data for analysis.

A safety committee of independent scientists not involved in the trial will analyse safety when half the intended accrual has been reached.

### Statistical Analysis

The only prospective study of the laparoscopic lavage operation so far reported (n = 92) a 1% occurrence of further surgery after the initial episode, whereas the other seven studies, all retrospective, varied between 0% and 100% reoperation, all of which were colon resections [18]. In our retrospective study [7] we found that 40% of the patients were re-operated for the same disease within a year, many of which were stoma reversals. To be able to detect a reduction from 40% to 10% in reoperations, each group must include at least 32 patients. The calculation is based on a binomial approximation, 80% power and a level of significance of 5%. In view of the relatively complicated flow chart for the trial and that all procedures are emergency surgery; the inclusion is set to 80 randomised patients (40 + 40).

Randomisation to blocks was generated using the Analysis Tool Pack in Excel, by the trial statistician. For each participating centre one block of 10 is sent at a time. The package consists of 10 closed envelopes, with numbers 1-10 on the outside, to be opened in sequence as the randomizations occur.

### Participating Hospitals

All participating centres are hospitals with emergency rooms and where abdominal surgery is performed.

The participating hospitals thus far are Sahlgrenska University Hospital, NU Hospital Organisation, Örebro University Hospital, Skövde Hospital/KSS, Alingsås Hospital, Central Hospital Karlstad, Herlev University Hospital, Roskilde University Hospital and Odense University Hospital. Six additional hospitals are in the process of becoming a participating institution.

### Ethics

The trial has been approved by the Danish (Protocol nr. H-4-2009-088) and the Swedish (EPN/Göteborg Dnr 378-09) ethics committees. In the Danish participating centres, the surgeon must receive informed consent from the patient.

In the Swedish participating centres, the surgeon can include the patient after informing a relative, if the patient's awareness is compromised by a septic condition.

## Discussion

Perforated diverticulitis requiring surgery is a potentially lethal condition with high risks regarding morbidity and mortality [8]. Although surgical techniques as well as results have developed considerably since Hartmann's Procedure was presented in 1921 [23], no significant changes in operative approach have been proven superior, for perforated diverticulitis Hinchey grade III-IV. Not only is the primary procedure associated with complications, but so is the reversal procedure [9]. Thus the combined risk of treatment for the patient is high. Diverticular disease is in general a condition associated with a considerable resource consumption [24], especially as the severity and quantity of complications lead to increased costs. It would therefore be beneficial for both patient and society if an alternative surgical technique could be introduced to minimise re-operations, re-admissions and permanent stomas. The recently described technique of laparoscopic lavage as treatment for perforated diverticulitis with peritonitis (Hinchey III) could be such an alternative treatment modality, but it remains to be validated in randomised trials. At present we are aware of at least three more randomised trials, the Scandiv study based at Department of Gastroenterological Surgery, Akershus University Hospital, Oslo [25], the Ladies trial based at Department of Surgery, Academic Medical Centre, Amsterdam [26], and the LapLAND study based at St. Vincent's University Hospital in Ireland [27].

The aim of the DILALA trial is to evaluate if laparoscopic lavage is a safe, minimally invasive method for patients with perforated diverticulitis Hinchey grade III, resulting in fewer re-operations, decreased morbidity, mortality, costs and increased quality of life. Inclusion started February 1^st ^2010 and by March 2011 eight hospitals had included patients, another three hospitals had gone through the steps to start inclusion.

The inclusion period is intended to be concluded by 2013.

## Acronym and Abbrevations

Diverticulitis Laparoscopic Lavage vs. Resection: DILALA; Laparoscopic lavage: LL; Hartmann's Procedure: HP; Quality of life: QoL; Clinical record forms: CRF

## Competing interests

The authors declare that they have no competing interests.

## Authors' contributions

EH is the principal investigator, has initiated the trial, been part of the planning of the trial, as well as the protocol writing committee. JR is the deputy principal investigator, has been part of the planning of the trial, as well as the protocol writing committee.

AT, EA, PJ, ZL, HO and SS have been part of the planning of the trial, as well as the protocol writing committee. AT and EA are also doctoral respectively post doctoral students, actively working with the trial. Registered Nurses EG and JH have both been part of the planning of the trial, visited participating hospitals to initiate the trial, created the database and are responsible for data collection and the logistics of the trial. All authors have been actively writing and reviewing the manuscript.

## Disclosure

All authors have agreed to the submission and have participated both in the study design and in the preparation of this manuscript.
